# Real-World Data on Treatment Management and Outcomes of Patients with Newly Diagnosed Advanced Epithelial Ovarian Cancer in Greece (The EpOCa Study)

**DOI:** 10.3390/curroncol28060440

**Published:** 2021-12-10

**Authors:** Michalis Liontos, Eleni Timotheadou, Emmanuel I. Papadopoulos, Zafeiris Zafeiriou, Dimitra Ioanna Lampropoulou, Gerasimos Aravantinos, Dimitrios Mavroudis, Christos Christodoulou, Adamantia Nikolaidi, Alvertos Somarakis, Christos Papadimitriou, Christos Papandreou, Aristotelis Bamias

**Affiliations:** 1 Department of Clinical Therapeutics, Alexandra General Hospital, School of Medicine, National and Kapodistrian University of Athens, 11528 Athens, Greece; mliontos@gmail.com; 2 Department of Medical Oncology, Papageorgiou Hospital, School of Medicine, Aristotle University of Thessaloniki, 56429 Thessaloniki, Greece; timotheadou@auth.gr (E.T.); cpapandr@otenet.gr (C.P.); 3 Medical Department, AstraZeneca, 15123 Athens, Greece; manos.papadopoulos@astrazeneca.com (E.I.P.); alvertos.somarakis@astrazeneca.com (A.S.); 4 Second Department of Medical Oncology, Theageneion Anticancer Hospital, 54007 Thessaloniki, Greece; zafeiris.zafeiriou@outlook.com; 5 Second Department of Medical Oncology, General Oncology Hospital of Kifissia “Agioi Anargiroi”, 14564 Athens, Greece; d_lambropoulou@yahoo.gr (D.I.L.); garavantinos@yahoo.gr (G.A.); 6 Department of Medical Oncology, University General Hospital of Heraklion, 71500 Heraklion Crete, Greece; mavroudis@uoc.gr; 7 Laboratory of Translational Oncology, Medical School, University of Crete, 71110 Heraklion Crete, Greece; 8 Second Department of Medical Oncology, Metropolitan Hospital, 18547 Piraeus, Greece; c_christodoulou@yahoo.gr; 9 Oncology Department, Mitera Hospital, 15123 Athens, Greece; MNikolaidi@mitera.gr; 10 Oncology Unit, 2nd Department of Surgery, ARETAIEIO University Hospital, School of Medicine, National and Kapodistrian University of Athens, 11528 Athens, Greece; cpapadim@med.uoa.gr; 11 2nd Propaedeutic Department of Internal Medicine, ATTIKON University Hospital, National and Kapodistrian University of Athens, 11527 Athens, Greece

**Keywords:** real world data, advanced ovarian cancer, treatment sequence, management strategy

## Abstract

New treatment modalities have been recently introduced in the management of ovarian cancer (OC). Herein, we sought to investigate their implementation in routine clinical practice and examine the real-world management of OC in Greece. EpOCa was a non-interventional, multicenter, retrospective study in patients with advanced epithelial OC. The primary outcome was to estimate the proportions of the different treatment regimens used per line of therapy, while progression-free survival (PFS) and overall survival (OS) were the key secondary endpoints. A total of 154 patients were enrolled in the study, among whom, 40% were tested for *BRCA* mutations and 30% were found to be positive. Nearly 90% of patients underwent debulking surgery at diagnosis, with few operations being also recorded upon relapse. Platinum-based chemotherapy (CT) was predominantly used in the first line with half of patients also receiving angiogenesis inhibitor (AI), while non-platinum-based CT was preferred in later lines. The median PFS was 18.2 and 8.8 months in the first- and second-line setting, respectively, whereas the median OS was approximately 50 months. Our study adds to the available, but limited, real world data on the management of ovarian cancer providing evidence regarding the applied treatment strategies and outcomes of patients in Greece.

## 1. Introduction

Epithelial ovarian cancer (EOC) is the most lethal gynecological cancer, representing the seventh most commonly diagnosed cancer among women in the world, with a 5-year survival rate of 44–46%. Annually worldwide, 295,000 women will be diagnosed and 184,000 will die. One of the main factors contributing to the high death-to-incidence rate is the advanced stage of the disease at the time of diagnosis [1,2,3]. In the newly-diagnosed advanced EOC (aEOC) setting, the current standard-of-care treatment involves primary debulking surgery followed by platinum- and taxane-based combination chemotherapy (CT) which, despite high initial response rates, eventually results in disease recurrence in most cases [4,5,6,7].

EOC is probably the tumor type with the highest percentage of hereditary cases. Pathogenic germline variants in *BRCA1/2* are responsible for the largest part of hereditary ovarian cancer. Beyond *BRCA1/2*, additional genes are suspected to participate in ovarian carcinogenesis [8,9].

Recently, an increased understanding of the molecular pathogenesis of EOC has led to molecularly targeted strategies designed to shift disease management towards more tailored therapeutic interventions [10,11]. Among them, the introduction of angiogenesis inhibitors (AIs) and, more recently, poly(ADP-ribose) polymerase inhibitors (PARPis) represent the most important advance [12]. Olaparib was the first PARPi approved for *BRCA* mutated OC by both the US Food and Drug Administration and the European Medicines Agency [13].

Although new systemic and surgical treatment modalities for OC have been introduced into practice based on evidence gathered from randomized controlled trials, there is limited data on the implementation of these interventions in the real world [14]. In Greece, the only evidence on management patterns and their outcomes in EOC comes from a large retrospective study on 1791 EOC cases diagnosed between 1976 and 2006. The study concluded that there were no significant differences in EOC patient characteristics and treatment patterns in Greece compared with those reported in the literature [15]. Since then, however, major therapeutic advances in aEOC management have occurred. In addition, registrational trials that led to regulatory approval of new agents in EOC contain constantly stricter inclusion criteria. In that setting, real world data provide evidence regarding the generalizability of randomized clinical trials data in the general population as well as a bridge to the knowledge gap between efficacy and effectiveness needed to satisfy Health Technology Assessment (HTA) bodies and regulatory authorities [16,17].

The present study represents an attempt to bridge this information gap, by examining the real-world management patterns, their sequencing and outcomes during the course of aEOC, in order to complement the evidence generated in the strictly controlled clinical trial setting, as well as to reveal factors that could guide treatment-decision making in the daily clinical practice for this heterogeneous and difficult-to-treat population.

## 2. Materials and Methods

EpOCa was a non-interventional, national, multicenter, retrospective medical chart review study. The study received independent ethics approval by the Institutional Review Boards (IRBs) of all participating hospitals. Deceased patients were only included in the study if a waiver of consent had already been granted by the IRBs of the participating sites. Chart abstraction initiated in November 2018 and completed by September 2019. Medical charts were reviewed and assessed through a process of consecutive sampling that follows reverse chronological order based on the date of initial diagnosis.

The study was carried out by hospital-based oncologists who treat patients with OC and practice in major anticancer hospitals in geographically diverse locations throughout Greece. Eligibility included patients who were >18 years old, had histologically documented advanced (Federation of Gynecology and Obstetrics [FIGO] stage III–IV) EOC, and were newly diagnosed between 1 September 2013 and 1 March 2016. Furthermore, eligible patients had to have sufficient available medical records for data abstraction to meet the objectives of the study, i.e., the patient had to be under the medical care of the participating site for the entirety of the patient observation period or the patient’s detailed historical data on their disease course, clinical management, and outcomes needed to be available at the participating site. As our study aimed at generating real-world data on management practices employed in routine care, subjects participating or who had participated since their aEOC diagnosis and throughout their data abstraction period, in any investigational trial with interventions outside of routine clinical practice were excluded. Patients satisfying the above criteria could be enrolled in this study following the provision of a written informed consent for collecting and processing medical data pertinent to study objectives unless a waiver of consent has been granted by the IRB of the study site.

The primary outcome of the study was the proportion of patients receiving each treatment regimen per line of therapy and the proportion of patients treated with unique treatment regimens sequence. The secondary outcomes were: the physician-defined progression-free survival (PFS), overall and by treatment regimen, in the first- and second-line therapeutic setting of aEOC from first/second-line treatment onset until the earliest date of physician-defined disease progression or death, the overall survival (OS) as of the time of treatment onset in the first-line therapeutic setting, and the physician-defined overall response rate (ORR) in the first- and second-line therapeutic setting of aEOC, overall and by treatment regimen. Furthermore, the study investigated any potential disease- and patient-related factors that guided the real-world treatment decision-making in the first- and second-line setting of aEOC in Greece. The variables collected for investigation as potential disease- and patient-related factors guiding treatment decision were: patient’s age at aEOC diagnosis, FIGO stage (III versus IV), tumor grade (low grade [G1]/high grade [G2, G3]) and histology (serous/non-serous), molecular testing history including *BRCA1/2* status, patient’s performance status (PS), symptoms and comorbidities, sites of metastasis, platinum-free interval of previous treatment line, type of previous therapy and residual disease status following cytoreductive surgery.

The study was descriptive in nature and was not planned to reject or affirm any formal statistical hypothesis. The sample size determination was conducted though to ensure that the descriptive data required for the study primary objective were sufficiently precise to draw meaningful and valid conclusions at a study level. Due to the scarcity of published real-world data pertaining to the aEOC management patterns in Greece, the worst-case scenario from a statistical viewpoint was considered for sample size calculation, corresponding to the assumption that any of the management strategies comprising the study primary endpoint may be observed in 50% of the patient population. According to this assumption, it was estimated that 100 patients was the minimum required sample size to provide a precision of estimation <10%. All of the descriptive analyses were performed in the overall eligible study population with available data and in the protocol-defined subpopulations of patients. Time-to-event analyses were performed using the Kaplan-Meier (KM) method. The associations of patient and disease characteristics with first- and second-line treatment choice were evaluated by binary and multinomial logistic models, as applicable. In the multivariable analyses, the factors examined in the univariable regression were included in the initial step of a stepwise procedure based on the minimization of Akaike’s Information Criterion. All of the statistical analyses were performed using SAS^®^ software, Version 9.4 of the SAS^®^ System for Windows [SAS Institute Inc., Cary, NC, USA].

## 3. Results

**Figure 1:**
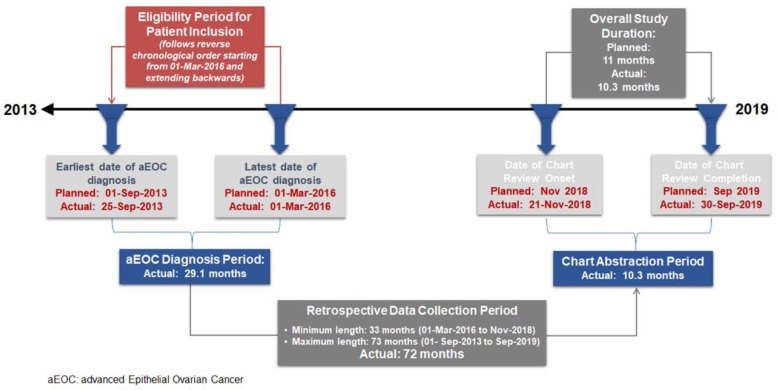
Retrospective chart review flow chart.

Over a 10.3-month chart abstraction period, from 21 November 2018 to 30 September 2019, a total of 163 patients were screened and enrolled in the study. Of these patients, 154 were eligible and included in the analysis. Eligible patients had been diagnosed with aEOC between 25 September 2013 and 1 March 2016 (Figure 1).

At the time of medical chart abstraction onset, 49.4% (76/154) of the patients were alive, while 50.6% (78/154) were deceased. The median (interquartile range) retrospective look-back period, defined as the time elapsed from aEOC diagnosis to death for deceased patients or to chart abstraction onset for patients alive at chart abstraction onset, was 43.1 (26.9–51.1) months. 

### 3.1. Patient and Disease Characteristics at aEOC Diagnosis

At the time of diagnosis, the median (range) age of the patients was 60.0 (21.0–90.0) years, while 74.0% (114/154) of them had been diagnosed with stage III and 26.0% (40/154) with stage IV EOC, according to FIGO classification. The primary tumor was well differentiated for 4.6% (6/131) of the evaluable patients, moderately differentiated for 16.0% (21/131), and poorly differentiated for 79.4% (104/131), while histological subtype was serous for the majority of the evaluable patients accounting for 83.3% (110/132). Moreover, 23.7% (28/118) of the evaluable patients had a family history of breast cancer or OC in first-degree relatives. Between aEOC diagnosis and chart abstraction, 40.9% (63/154) of the patients had undergone a total of 71 molecular tests for reasons related to EOC. Of the 63 patients that underwent at least one test, 62 patients (40.3%) were tested for *BRCA1* and/or *BRCA2*, of whom 30.6% (19/62) were *BRCA*-mutated. Specifically, 32 of the 62 (51.6%) patients were tested for *BRCA1* and/or *BRCA2* between aEOC diagnosis and first disease progression, 27 (43.5%) patients were tested at or after first disease progression, and 3 (4.9%) patients were tested twice both before and at or after first disease progression with test results being consistent between the two time points for all 3 patients. The main baseline characteristics of the patients are summarized in Table 1.

### 3.2. Surgical Management since aEOC Diagnosis

Following the initial diagnosis, 90.3% (139/154) of the patients had been subjected to cytoreductive surgery for the management of aEOC, while the rest 9.7% (15/154) of the patients had no surgery. Among them (*n* = 139), 58.3% (81/139) underwent primary debulking surgery, 38.1% (53/139) of the patients had interval debulking surgery, while the remaining five patients (3.6%) received preoperative CT and then subjected to debulking surgery without receiving postoperative CT. The outcome of the surgery could not be retrieved in the medical records of the 83 out of the 139 patients (59.7%) subjected to debulking surgery. For the other 56 patients (40.3%), the outcome of surgery was: complete resection of all macroscopically visible disease in 58.9% (33/56) of the cases, optimal resection but with visible residual disease (0.1–1.0 cm in maximal diameter) in 19.7% (11/56), and suboptimal resection (residual disease >1.0 cm) in the 21.4% (12/56) of the cases. In addition, 9.1% (14/154) of the patients underwent secondary debulking surgeries. Specifically, 12 surgeries were performed at the first disease progression while the other 2 surgical operations were performed at the second disease progression (Table S1).

### 3.3. Systemic Therapy Strategies and Sequencing Patterns throughout the Course of the Disease

All 154 eligible patients had received first-line systemic therapy. With regards to the subsequent lines of treatment, 70.8% (109/154) of the patients had received second line, 48.1% (74/154) third line, and 31.2% (48/154) fourth line, while a small percentage of patients had received up to seven lines of therapy.

Although platinum-based CT was the preferable choice of treatment in the first- (100%) and second- (66.1%) line settings, rates of platinum-based CT use decreased as patients progressed through treatment line, to the benefit of non-platinum-based regimens (Table S1).

The first-line management strategy included CT alone in 52.7% and CT plus AI in 47.3% of the patients. AI was also used in less than 16% of patients in later lines of therapy. PARPi administration was recorded in all 19 patients tested and found to be positive for *BRCA1* and/or *BRCA2* mutations, with most patients having received PARPi in the second (11.9%) line. The most common first- to second-line treatment patterns (at a frequency >10%) were platinum-based CT plus AI followed by platinum-based CT (24.8%; 27/109), platinum-based CT alone followed by another platinum-based CT regimen (20.2%; 22/109), platinum-based CT followed by non-platinum-based CT (19.3%; 21/109) and platinum-based CT plus AI followed by platinum-based CT plus targeted therapy (TT) (12.8%; 14/109); TT included PARPi in 8 patients, AI in 5 patients, and AI + PARPi in 1 patient. Both in first- and second-line treatment, AI corresponded to bevacizumab and PARPi to olaparib. The treatment patterns and combinations of agents varied after the third line of treatment (Table S1).

### 3.4. PFS and ORR in the First- and Second-Line Treatment Settings

#### 3.4.1. PFS and ORR in the First-Line Setting

**Figure 2:**
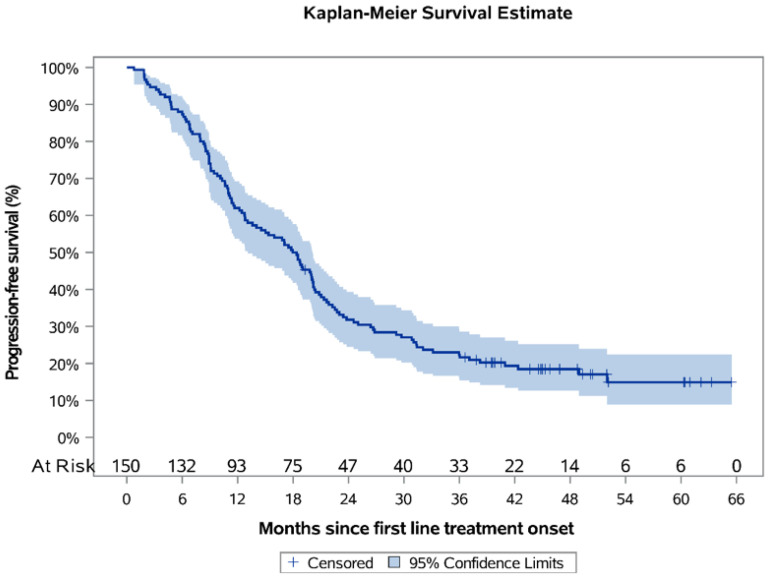
Kaplan-Meier progression-free survival curves in the first-line treatment setting.

**Figure 3:**
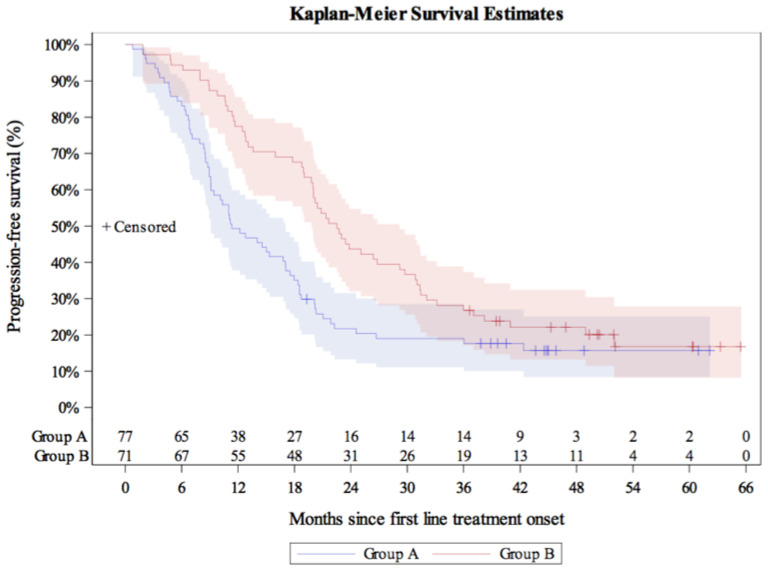
Kaplan-Meier progression-free survival curves in the subgroups of patients who had received first-line platinum-based CT alone (Group A) and platinum-based CT plus angiogenesis inhibitor (Group B).

Over a median KM-estimated 48.79 months of follow-up since postoperative systemic treatment onset, the 12-month PFS rate was estimated to be 62.0% (95% CI: 53.7–69.2), and the estimated median PFS was 18.2 months (95% CI: 13.1–20.1) (Figure 2). For the subpopulations who had received platinum-based CT only (*N* = 77) (median duration of therapy 3.5 months) and platinum-based CT + bevacizumab (*N* = 71) (median duration of therapy 11.7 months), the median PFS was estimated to be 11.3 months (95% CI: 9.1–17.0) and 22.5 months (95% CI: 19.8–29.2), correspondingly (Figure 3), while the respective KM-estimated 12-month PFS rates were 49.4% (95% CI: 37.8–59.9) and 77.5% (95% CI: 65.9–85.5). 

The ORR for the overall population was 48.9% (45/92; 95% CI: 38.7–59.1), while the ORR for the subpopulations who had received platinum-based CT only and platinum-based CT + bevacizumab was 44.4% (20/45) and 54.3% (25/46), respectively. 

#### 3.4.2. PFS and ORR in the Second-Line Setting

**Figure 4:**
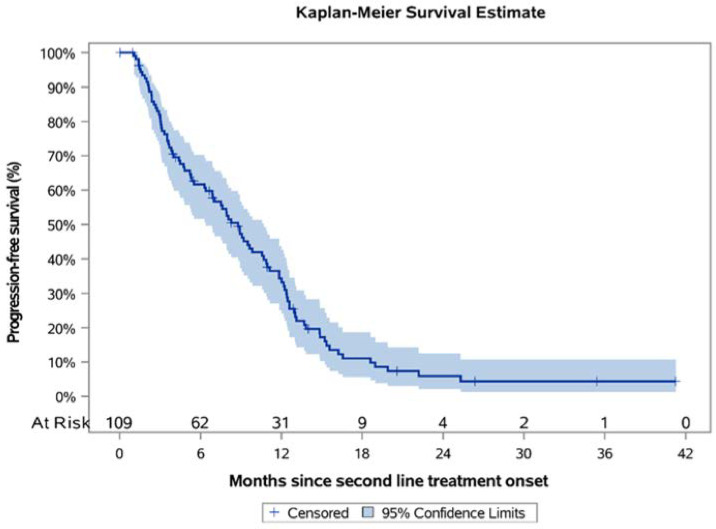
Kaplan-Meier progression-free survival curve in the second-line treatment setting.

Over a median KM-estimated 26.36 months of follow-up since second-line treatment onset, the 12-month PFS rate was 33.2% (95% CI: 24.0–42.6) and the estimated median PFS was 8.8 months (95% CI: 6.4–10.7) (Figure 4), while the ORR was 41.4% (36/87; 95% CI: 31.0–51.7). Among patients that progressed following first-line treatment, 68.8% (75/109) had platinum-sensitive disease, while 31.2% (34/109) had platinum-resistant/refractory disease. For these subpopulations, the median PFS was estimated to be 11.0 (95% CI: 8.3–12.4) and 3.6 (95% CI: 2.4–6.4) months, correspondingly, while the respective KM-estimated 12-month PFS rates were 42.2% (95% CI: 30.0–53.9) and 14.7% (95% CI: 5.4–28.5) (Figures S1 and S2). Furthermore, there was a subpopulation of patients with *BRCA* mutations that all received olaparib in the second-line setting (*N* = 11). The median PFS for these patients was estimated to be 19.0 (95% CI: 8.9-NR) months with the 12-month PFS rate being 66.7% (95% CI: 28.2–87.8). It is worthwhile mentioning that olaparib was also administered in two patients whose *BRCA* status was unknown upon chart review. When these two patients were added in the analysis, the mPFS did not differ from the one reported above (19.0; 95% CI: 9.2–NR).

### 3.5. Overall Survival

**Figure 5:**
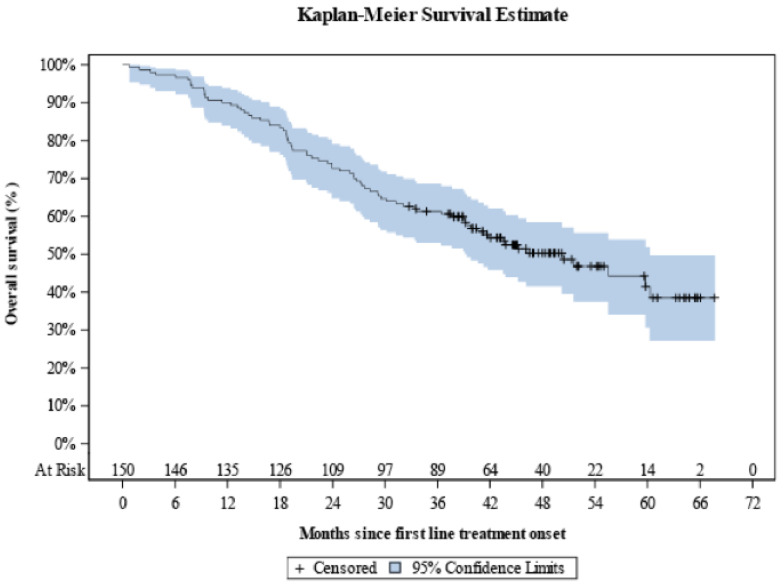
Kaplan-Meier overall survival curve since first-line treatment onset in the overall population.

Over a median KM-estimated 48.7 months of follow-up since first-line treatment onset, the median OS was 50.2 months (95% CI: 39.4–60.3) with the 12-month OS rate being equal to 90.0% (95% CI: 84.0–93.8), and the respective 24-, 36-, 48- and 60-month rates being calculated to be 72.7% (95% CI: 64.8–79.1), 61.3% (95% CI: 53.0–68.6), 50.3% (95% CI: 41.6–58.4), and 41.5% (95% CI: 30.7–51.9) (Figure 5).

### 3.6. Association of Patient and Disease Characteristics with First- and Second-Line Treatment Choice

According to the results of univariate analysis, age <65 years and FIGO stage III at aEOC diagnosis were identified as being positively associated with the receipt of platinum-based CT + bevacizumab vs platinum-based CT only, whereas ECOG PS ≥ 1, malignant pleural effusion/lung/pleura metastasis, extra-abdominopelvic metastases and residual disease following cytoreductive surgery were found to be negatively associated with the receipt of platinum-based CT + bevacizumab vs platinum-based CT only in the first-line setting. However, these correlations could not be further evaluated though multivariable analysis, as FIGO stage and age were the only parameters included in the relative multivariable model with several other factors of interest being excluded due to a data missing rate exceeding 10% (Table S2). In the second-line setting, the association of selected patient and disease characteristics with the choice of different treatment patterns could not be appropriately performed due to the heterogeneity and complexity of the data that meant patients were divided into several different groups according to platinum-free interval and administered regimens.

## 4. Discussion

Our study objective was to evaluate the implementation and clinical outcomes of systemic and surgical treatment modalities in patients with advanced OC in a real-world setting in Greece. According to our data, platinum-based CT was received by all patients in the first-line setting, while its use decreased as patients advanced to subsequent treatment lines, where non-platinum-based CT was the preferred regimen. Most patients (90%) underwent primary or interval debulking surgery with few patients having been also subjected to surgery upon relapse. Interestingly, though, the outcome of the surgery was unknown in the majority of the recorded cases. In addition, approximately one-half and one-quarter of the patients were treated with CT plus TT in the first and second line of therapy, respectively, comprising bevacizumab in all cases of the first-line setting, and either olaparib or bevacizumab in the second one. Overall, these results indicated that the management strategies and sequencing patterns applied in Greece were similar to those reported in other countries [18,19]. More specifically, a multi-country survey conducted across the USA and in four European countries (France, Italy, Germany, UK) concluded that patients diagnosed with advanced OC from December 2016 to January 2017 were largely treated with first-line platinum-based CT only in all countries but Germany, where physicians predominantly administered bevacizumab in combination with CT. In the same study, the use of platinum-based CT seemed to decrease as patients recurred, with a switch towards non-platinum-based CT [18]. Towards the same direction, an international, multi-center retrospective medical chart review of 2100 randomly selected patients diagnosed with Stage III-IV OC and actively receiving anti-cancer treatment during chart review period was conducted between July 2016 and June 2017 in the USA and five European countries (France, Germany, Italy, Spain, UK). The main conclusion drawn from this study was that the carboplatin-paclitaxel combination was both the most common regimen used overall and the most common first-line administered therapy. Moreover, among patients receiving second-line regimens, liposomal doxorubicin was the most commonly used drug in the USA in comparison to bevacizumab, which was predominantly administered in Europe [19]. Regarding the proportion of patients tested for *BRCA* mutations, data were provided by the study of Audibert et al., who reported that the relative proportions were 45% in Italy, 50% in the UK, 61% in France, 65% in Germany, and 73% in the USA. Of the patients tested for *BRCA* mutations, 10% had a positive test in the UK, 13% in France and Germany, 16% in the USA, and 21% in Italy [18]. In our study, the proportion of *BRCA*-tested patients was 40% among whom 30% were BRCA-mutated. During the data collection period, testing for BRCA mutations was not reimbursed in Greece. Despite ESMO guidance for molecular testing in all newly diagnosed aEOC patients is adopted by Greek physicians, limitations in reimbursement justify the percentage recorded in this study.

With regards to clinical outcomes, the median PFS estimated for first- and second-line treatment setting was equal to 18.2 and 8.8 months, respectively, whereas the median OS was approximately 50 months. These numbers highlight the need to develop more effective and individualized treatment options for OC patients. Focusing on first-line setting, the estimated median PFS favored the combination of platinum-based CT plus bevacizumab vs CT only. In terms of efficacy, these results were in line with those generated within the context of GOG 0218 phase III trial where the addition of bevacizumab to upfront intravenous CT provided a median PFS improvement vs placebo [20]. In second-line setting, median PFS was shorter than the one estimated in the first-line treatment, while as also expected, patients with platinum-sensitive relapse had a longer median PFS compared to those with platinum-resistant disease. In addition, patients with *BRCA* mutations appeared to derive clinical benefit from the addition of the PARPi olaparib, although the number of analyzed patients was small enough to draw safe conclusions. 

A number of factors that seemed to have guided physicians’ treatment decisions were also identified by our study. Specifically, it appeared that Greek Medical Oncologists applied the therapeutic strategy of adding bevacizumab to first-line platinum-based CT, to younger patients and those with more favorable prognostic features, whereas patients with a more “high-risk” phenotype shared less probability of receiving this drug. This strategy appears to be controversial to what has been shown in the registrational clinical trials ICON-7 and GOG-0218 of bevacizumab, where significant PFS benefit was recorded in the poor-prognosis patients [20,21]. However, due to restrictions imposed by the insufficient amount of data of some clinically important parameters, all aforementioned significant associations were generated through univariable analysis only and therefore all results need to be interpreted with caution. In the second-line setting, the complexity of the data sets and the number of available observations per defined group did not allow us to perform an analogous comprehensive statistical analysis. Nevertheless, a descriptive statistical analysis was conducted revealing that treating physicians chose to administer non-platinum-based CT in the 16% of the patients with platinum-sensitive relapse. 

Our study has limitations that can be attributed to its retrospective design and observational nature mainly including patient selection, confounding, and information bias. Measures and steps were taken to address these limitations and mitigate them. Such measures included consecutive, thus non-selective, enrollment of the first eligible patients, the availability of sufficient relevant medical records and thorough cleaning session of the data prior to analysis, and source data verification. Nevertheless, due to the inherent limitations of retrospective analysis, we could not prevent issues related to missing data in a few cases concerning important prognostic indicators, such as the outcome of debulking surgeries, which reflects, though, the frequent underreporting of residual disease in surgical reports in some sites. Furthermore, the study population was enrolled from sites located in 3 of the 13 administrative regions of Greece, which are home to 58% of the overall Greek population. However, despite the limited geographic diversity, the eight oncology hospital centers participating in EpOCa represent some of the major oncology clinics of the country, aiding the generalizability of the study results. Another possible limitation of our study is the fact that no safety data were collected. However, since EpOCa was a retrospective study based on data abstraction from medical records and subsequent recording of only drug classes and not active substances, a collection of safety data was estimated to be not applicable.

## 5. Conclusions

In conclusion, our study is the first RWE study in Greece that attempts to bridge the information gap between the real-world management patterns and the evidence generated in the strictly controlled clinical trial setting. Despite its retrospective design and any limitations, our study met its objectives by documenting the overall management strategies followed in aEOC and their survival outcomes. Following their approval, novel targeted treatments have been incorporated into the management of the disease, while molecular testing has been adapted to an extent analogous with international standards. Due to local differences in drug reimbursement and availability of molecular testing such RWE studies could provide useful insight for the management of this difficult-to-treat population.

## Figures and Tables

**Table 1:** Patient characteristics.

Patient Characteristics at aEOC Diagnosis Eligible Population: *n* = 154		
Age (years)	npt	154
	Mean (SD)	60.1 (13.7)
	Median	60.0
	Min-Max	21.0–90.0
CA-125 levels (U/m)	npt	82
	Mean (SD)	1293.5 (2086.7)
	Median	424.5
	Min-Max	21.0–11240.0
Serum albumin levels (g/dL)	npt	31
	Mean (SD)	3.8 (0.7)
	Median	3.8
	Min-Max	2.3–4.8
FIGO stage	III	114 (74.0%)
	*IIIA*	6 (3.9%)
	*IIIB*	15 (9.7%)
	*IIIC*	89 (57.8%)
	*Unknown*	4 (2.6%)
	IV	40 (26.0%)
	*IVA*	12 (7.8%)
	*IVB*	21 (13.6%)
	*Unknown*	7 (4.5%)
Tumor grade	G1: well differentiated	6 (3.9%)
	G2: moderately differentiated	21 (13.6%)
	G3: poorly differentiated	104 (67.5%)
	Unknown	23 (14.9%)
Histological subtype	Serous	108 (70.1%)
	Unspecified adenocarcinoma	15 (9.7%)
	Endometrioid	8 (5.2%)
	Mixed type	7 (4.5%)
	Mucinous	5 (3.2%)
	Clear cell	2 (1.3%)
	Other	9 (5.6%)
ECOG PS	0	53 (34.4%)
	1	23 (14.9%)
	2	4 (2.6%)
	3	3 (1.9%)
	Unknown	71 (46.1%)

Abbreviations: aEOC: advanced epithelial ovarian cancer; CA-125: cancer antigen 125; ECOG PS: Eastern Cooperative Oncology Group performance status; FIGO: Federation of Gynecology and Obstetrics; npt: number of patients with available observations; SD: standard deviation.

## Data Availability

The datasets used and/or analyzed during the current study are available from the corresponding author on reasonable request.

## Supplementary Materials

The following are available online at https://www.mdpi.com/article/10.3390/curroncol28060440/s1, Figure S1: Kaplan-Meier progression-free survival curve of patients with platinum-sensitive relapse in the second-line setting. Figure S2: Kaplan-Meier progression-free survival curve of patients with platinum-resistant/refractory relapse in the second-line setting. Table S1: Summary of data collected for surgical management and systemic therapy of recruited patients. Table S2: Univariable and multivariable logistic regression analysis for the association of first-line treatment (platinum-based chemotherapy + bevacizumab versus platinum-based chemotherapy only) with factors of interest.
