# Supplementary material for: Real-World Data on Treatment Management and Outcomes of Patients with Newly Diagnosed Advanced Epithelial Ovarian Cancer in Greece (The EpOCa Study)
Source: Curr Oncol. 2021 Dec 10;28(6):5266–77. doi: 10.3390/curroncol28060440 (PMC8699844; doi:10.3390/curroncol28060440)
Supplement: Supplementary file 1 [file curroncol-28-00440-s001.zip › curroncol-1422073-supplementary.pdf]

Current Oncology

Real-World Data on Treatment Management and Outcomes of Patients with Newly Diagnosed Advanced Epithelial Ovarian Cancer in Greece (the EpOCa Study)

Michalis Liontos, Eleni Timotheadou, Emmanuel I. Papadopoulos, Zafeiris Zafeiriou, Dimitra Ioanna Lampropoulou, Gerasimos Aravantinos, Dimitrios Mavroudis, Christos Christodoulou, Adamantia Nikolaidi, Alvertos Somarakis, Christos Papadimitriou, Christos Papandreou and Aristotelis Bamias

Table S1. Summary of data collected for surgical management and systemic therapy of recruited patients.

Surgical procedures					
	**Before 1st PD (N = 139)**			**After 1st PD (N = 14)**	
	**Primary debulking**	**Interval debulking**	**Debulking Surgery following NACT only**	**Secondary debulking**	
				**At 1st PD**	**At 2nd PD**
	**n_{pt} (%)**	**n_{pt} (%)**	**n_{pt} (%)**	**n_{pt} (%)**	**n_{pt} (%)**
	81 (58.3%)	53 (38.1%)	5 (3.6%)	12 (85.7%)	2 (14.3%)
**Surgical outcomes**					
Complete resection of all macroscopic visible disease	18 (22.2%)	14 (26.4%)	1 (20.40%)	1 (8.3%)	1 (50.0%)
Optimal but visible residual disease (0.1-1.0 cm in maximal diameter)	7 (8.7%)	4 (7.6%)	-	-	-
Suboptimal debulking (residual disease >1.0cm)	7 (8.7%)	5 (9.4%)	-	1 (8.3%)	-
Unknown	49 (60.4%)	30 (56.6%)	4 (80.0%)	10 (83.3%)	1 (50.0%)

Systemic therapy							
	**1st line (N = 154)**	**2nd line (N = 109)**	**3rd line (N = 74)**	**4th line (N = 48)**	**5th line (N = 31)**	**6th line (N = 15)**	**7th line (N = 3)**
	**n_{pt} (%)**	**n_{pt} (%)**	**n_{pt} (%)**	**n_{pt} (%)**	**n_{pt} (%)**	**n_{pt} (%)**	**n_{pt} (%)**
Platinum-based CT	81 (52.7%)	49 (45.0%)	33 (44.6%)	11 (22.9%)	7 22.6%)	4 (26.7%)	1 (33.3%)
Platinum-based CT + angiogenesis inhibitor	73 (47.3%)	9 (8.3%)	1 (1.4%)	-	-	-	-

Platinum-based CT + PARP inhibitor	-	9 (8.3%)	1 (1.4%)	1 (2.1%)	-	1 (6.7%)	-
Non-platinum-based CT	-	31 (28.4%)	35 (47.2%)	33 (68.7%)	22 (71.0%)	8 (53.3%)	1 (33.3%)
Non-platinum-based CT + angiogenesis inhibitor	-	4 (3.7%)	2 (2.7%)	2 (4.2%)	1 (3.2%)	-	-
Other*	-	7 (6.3%)	2 (2.7%)	1 (2.1%)	1 (3.2%)	2 (13.3%)	1 (33.3%)

CT: chemotherapy; N: total number of patients; n_{pt}: number of patients per subgroup; NACT: neoadjuvant chemotherapy; PD: progression of disease; PARP: poly (ADP-ribose) polymerase.*In the 2nd line, 4 patients received platinum-based chemotherapy + angiogenesis inhibitor + PARP inhibitor, 1 patient received platinum compound + antiestrogen therapy, 1 patient received antiestrogen therapy, and 1 patient was treated with an aromatase inhibitor; in the 3rd line, 1 patient received non-platinum-based chemotherapy + PARP inhibitor and 1 patient was treated with PARP inhibitor monotherapy; in the 4th line, 1 patient received progestogen; in the 5th line, 1 patient received platinum-based chemotherapy + angiogenesis inhibitor + PARP inhibitor; in the 6th line, 1 patient received antiestrogen therapy and 1 patient was treated with angiogenesis inhibitor monotherapy; in the 7th line, 1 patient received aromatase inhibitor.

Table S2. Univariable and multivariable logistic regression analysis for the association of first-line treatment (platinum-based chemotherapy + bevacizumab versus platinum-based chemotherapy only) with factors of interest.

**Univariable logistic regression analysis<br>Eligible patients receiving Platinum-based CT + bevacizumab and platinum-based CT first-line treatment: *N*=148**							
					**95% CI**		
**Parameter**	**Category vs. reference**	**n_{pt}**	**n_{GroupB}**	**OR**	**Lower limit**	**Upper limit**	**p-value**
**Age at aEOC diagnosis (*N*=148)**	<65 vs ≥ 65 years	86 vs 62	51 vs 20	3.06	1.54	6.07	0.001
**FIGO stage at aEOC diagnosis (*N* = 148)**	III vs IV	110 vs 38	59 vs 12	2.51	1.15	5.47	0.021
**Tumor grade at aEOC diagnosis (*N* = 127)**	G3 vs G1/G2	102 vs 25	51 vs 8	2.12	0.84	5.36	0.111
**Tumor histology at aEOC diagnosis (*N* = 126)**	Serous vs Non-serous	106 vs 20	57 vs 6	2.71	0.97	7.60	0.057
**BRCA1/BRCA2 status between aEOC diagnosis and first disease progression (*N* = 35)**	Positive vs Negative	13 vs 22	9 vs 18	0.50	0.10	2.48	0.396
**ECOG PS at first-line treatment onset (*N* = 83)**	≥1 vs 0	23 vs 60	2 vs 32	0.08	0.02	0.39	0.002
**Presence of ascites at first-line treatment onset (N=116)**	Yes vs No	46 vs 70	19 vs 32	0.84	0.39	1.77	0.640
**Presence of symptoms at first-line treatment onset (*N* = 102)**	Yes vs No	30 vs 72	14 vs 32	1.09	0.47	2.57	0.837

**Presence of comorbidities at first-line treatment onset (*N* = 137)**	Yes vs No	84 vs 53	34 vs 28	0.61	0.30	1.21	0.158
**At least one site of disease localization at first-line treatment onset (*N* = 127)**	Yes vs No	67 vs 60	27 vs 34	0.52	0.25	1.05	0.067
**Lymph nodes metastases at first-line treatment onset (*N* = 127)**	Yes vs No	22 vs 105	11 vs 50	1.10	0.44	2.76	0.839
**Peritoneum metastasis at first-line treatment onset (*N* = 127)**	Yes vs No	35 vs 92	16 vs 45	0.88	0.40	1.92	0.748
**Malignant pleural effusion/lung/pleura metastasis at first-line treatment onset (*N* = 127)**	Yes vs No	22 vs 105	4 vs 57	0.19	0.06	0.59	0.004
**Extra-abdominopelvic metastases at first-line treatment onset (*N* = 127)**	Yes vs No	25 vs 102	6 vs 55	0.27	0.10	0.73	0.010
**Residual disease status following cytoreductive surgery prior to first-line treatment onset (*N* = 54)**	Yes vs No	23 vs 31	7 vs 18	0.32	0.10	0.99	0.047

Multivariable logistic regression analysis**

Eligible patients receiving Platinum-based CT + bevacizumab and platinum-based CT first-line treatment: N=148

**Parameter**	**Category vs. reference**	**OR**	**95% CI Lower limit**	**95% CI Upper limit**	**p-value**
**Number of observations used in model: *N* = 137**					
**FIGO stage aEOC diagnosis**	III vs IV	4.01	1.56	10.28	0.004
**Presence of comorbidities at first-line treatment onset**	Yes vs No	0.54	0.26	1.11	0.095

aEOC: advanced epithelial ovarian cancer; CI: confidence interval; CT: chemotherapy; ECOG PS: eastern cooperative oncology group performance status FIGO: International Federation of Gynecology and Obstetrics; N: total number of patients; n_{pt}: number of patients per subgroup; n_{GroupB}: number of patients per subgroup of Group B; OR: odds ratio. **The modeled probability was 'Platinum-based CT + bevacizumab (Group B)' vs. 'Platinum-based CT (Group A)' first line treatment regimen. *The following factors were included in the initial step of the stepwise procedure: age at aEOC diagnosis, FIGO stage at aEOC diagnosis, presence of comorbidities at first-line treatment onset. Factors examined in univariable analyses with a missing rate exceeding 10% were not included in the initial step of the stepwise procedure.

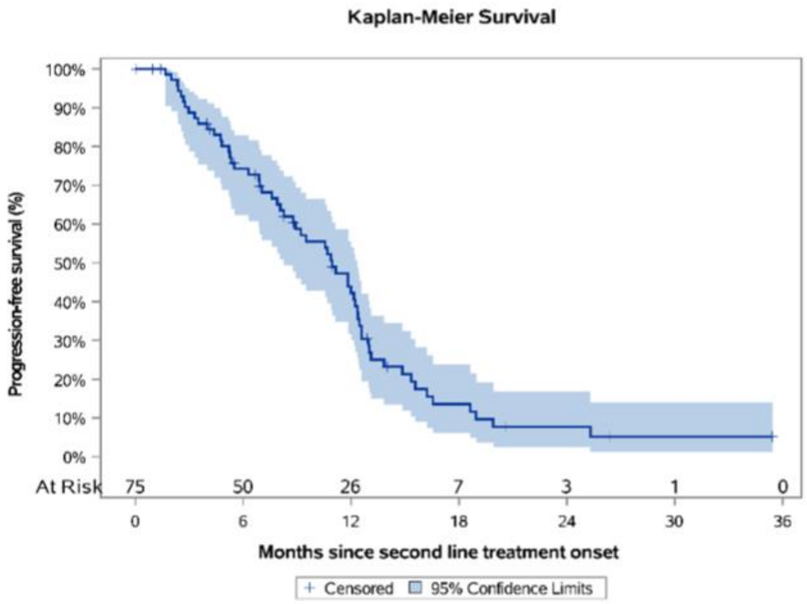

Figure S1. Kaplan-Meier progression-free survival curve of patients with platinum-sensitive relapse in the second-line setting.

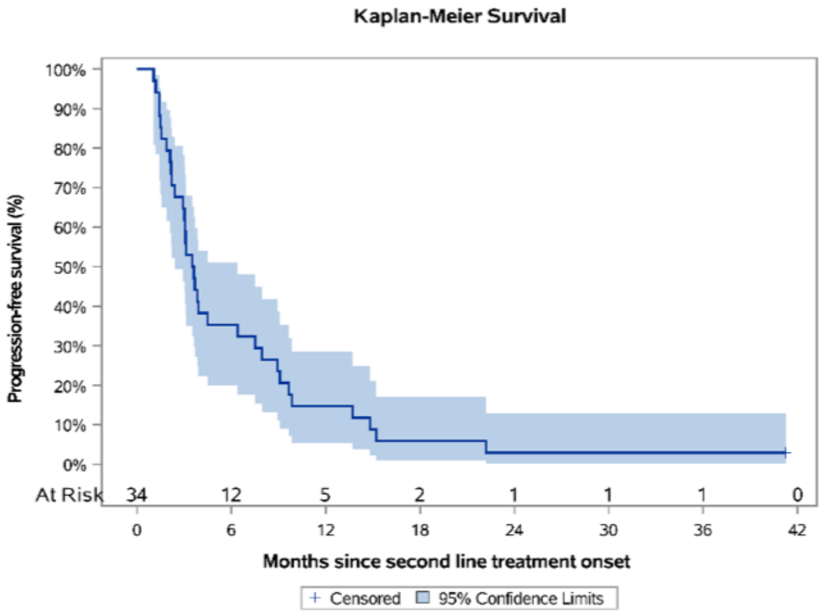

Figure S2. Kaplan-Meier progression-free survival curve of patients with platinum- resistant/refractory relapse in the second-line setting. .
